# The Role of Immersive Virtual Reality and Augmented Reality in Medical Communication: A Scoping Review

**DOI:** 10.1177/23743735231171562

**Published:** 2023-06-27

**Authors:** Ibrahim Aliwi, Vico Schot, Michele Carrabba, Phuoc Duong, Silvia Shievano, Massimo Caputo, Jo Wray, Adelaide de Vecchi, Giovanni Biglino

**Affiliations:** 1 Birmingham Medical School, Birmingham, UK; 2 152331 Bristol Medical School, University of Bristol, Bristol, UK; 3 Alder Hey Children's Hospital, Liverpool, UK; 4 UCL Institute of Cardiovascular Science, 4919 UCL, London, UK; 5 University Hospitals Bristol & Weston NHS Foundation Trust, Bristol, UK; 6 4956 Great Ormond Street Hospital for Children NHS Foundation Trust, London, UK; 7 Department of Biomedical Engineering, 4616 King's College London, London, UK; 8 National Heart and Lung Institute, Imperial College London, London, UK

**Keywords:** technology, clinician–patient relationship, communication, empathy

## Abstract

Communication between clinicians and patients and communication within clinical teams is widely recognized as a tool through which improved patient outcomes can be achieved. As emerging technologies, there is a notable lack of commentary on the role of immersive virtual reality (VR) and augmented reality (AR) in enhancing medical communication. This scoping review aims to map the current landscape of literature on this topic and highlights gaps in the evidence to inform future endeavors. A comprehensive search strategy was conducted across 3 databases (PubMed, Web of Science, and Embase), yielding 1000 articles, of which 623 were individually screened for relevance. Ultimately, 22 articles were selected for inclusion and review. Similarities across the cohort of studies included small sample sizes, observational study design, use of questionnaires, and more VR studies than AR. The majority of studies found these technologies to improve medical communication, although user tolerability limitations were identified. More studies are required, presenting more robust findings, in order to draw more definitive conclusions and stronger recommendations for use of immersive VR/AR in clinical environments.

## Introduction

Immersive virtual reality (VR) and augmented reality (AR) are gaining increasing attention within the medical field and have been extensively researched in relation to teaching and treatment applications. However, there is a notable lack of commentary on how these emerging technologies impact different types of communication within a healthcare context.

Immersive VR refers to a simulated virtual environment delivered to a user via visual, auditory, and sometimes haptic stimuli through a head-mounted display (HMD).^1^ The immersive aspect is derived from interactivity and tracking of the user's head movements, creating spatial presence within the virtual world.^2^ Augmented reality refers to the integration and superimposition of digital elements into the user's real-life environment, so that both can be attended to simultaneously.^3^ This is facilitated through different means and is most commonly enabled by smartphones and smart glasses.^4^

Communication is widely recognized as critical in the clinical setting, both when considering communication between clinicians and their patients and communication within clinical teams. For example, better quality doctor–patient communication yields increased patient understanding, treatment compliance, and satisfaction,^5-8^ thus having an overall direct impact on patient experience. Health Education England recognizes that “good communication skills have a positive effect on health outcomes”.^9^ Better communication can lead to increasing patients’ well-being and, according to the UK “Public Health Outcomes Framework” (2019-22), self-reported well-being is an indicator of health improvement, as is emotional well-being of looked after children. This supports people “to live healthy lifestyles, make healthy choices and reduce health inequalities.^10^”

Recently, VR has established exposure therapy use-cases in the treatment of psychiatric disorders such as phobias and anxiety.^11-13^ Other technologies such as 3D printing have also shown promise in the area of medical communication,^14^ which is suggestive of the potential VR and AR have in replicating this effect in the same communicative frame, as an alternative 3D visualization technology.

Thus, the aim of this scoping review was to map and evaluate the current landscape of studies researching immersive VR and AR interventions in medical communication, through a comprehensive search strategy and critical appraisal of the literature. Future research endeavors are recommended based on the resulting body of evidence.

## Methods

### Search Strategy

A literary search was conducted (July 2022) using 3 databases: PubMed, Web of Science, and Embase. Four Boolean logic search strategies were formulated and used in each database, including: “(((((((((Virtual Reality) OR (Augmented Reality)) OR (Mixed Reality)) OR (3D Technology)) OR (3D Technologies)) OR (Holography)) OR (3D Visualisation)) OR (Head Mounted Display)) AND (Doctor-Patient)) NOT (Printing),” “((((((((((Virtual Reality) OR (Augmented Reality)) OR (Mixed Reality)) OR (3D Technology)) OR (3D Technologies)) OR (Holography)) OR (3D Visualisation)) OR (Head Mounted Display)) AND (Counselling)) AND (Communication)) NOT (Printing),” “((((((((((Virtual Reality) OR (Augmented Reality)) OR (Mixed Reality)) OR (3D Technology)) OR (3D Technologies)) OR (Holography)) OR (3D Visualisation)) OR (Head Mounted Display)) AND (Counselling)) AND (Discussion)) NOT (Printing),” and “(((((((Virtual Reality) OR (Augmented Reality)) OR (Mixed Reality)) OR (Holography)) OR (Head Mounted Display)) AND (Training) AND (Patient)) AND (Communication)) NOT (Printing).” These terms were curated to yield a wide diversity of articles on medical communication by encompassing training, counseling, and discuss applications of VR/AR, as well as being expanded to include a variety of synonyms for 3D visualization technologies. 3D printing was excluded from the strategy as this was outside the scope of this review.

### Inclusion and Exclusion Criteria

The following exclusion criteria were applied to the results of the search: any type of review article (due to no original findings), conference proceedings or abstracts (to avoid duplicate findings if published as an article), and duplicate articles across different searches.

Inclusion criteria were that the study must be assessing an immersive VR intervention delivered through an HMD or headset, rather than a traditional virtual experience on a flat, non-immersive display. Augmented reality interventions inherently also use head-mounted devices, but other projection-based devices were also accepted. The study must also evaluate the effects on communication within a medical context (between doctor and patient, or between healthcare professionals within a team). Articles commenting on the tolerability or feasibility of using the technology in this context were also included.

A manual review of the remaining articles was performed (IA), a second party was consulted where necessary (GB), and a third party would intercede if a dispute arose over inclusion (VS).

The final articles were then screened, mapped in detail, and tabulated, to identify patterns and themes in the relevant literature. Critical appraisal was performed, with an emphasis on gauging types of VR/AR interventions used and establishing that a study indeed assessed or measured changes in communication or empathy or perceptions around the technology in a communicative setting (eg, not for surgical training).

**Figure 1.**
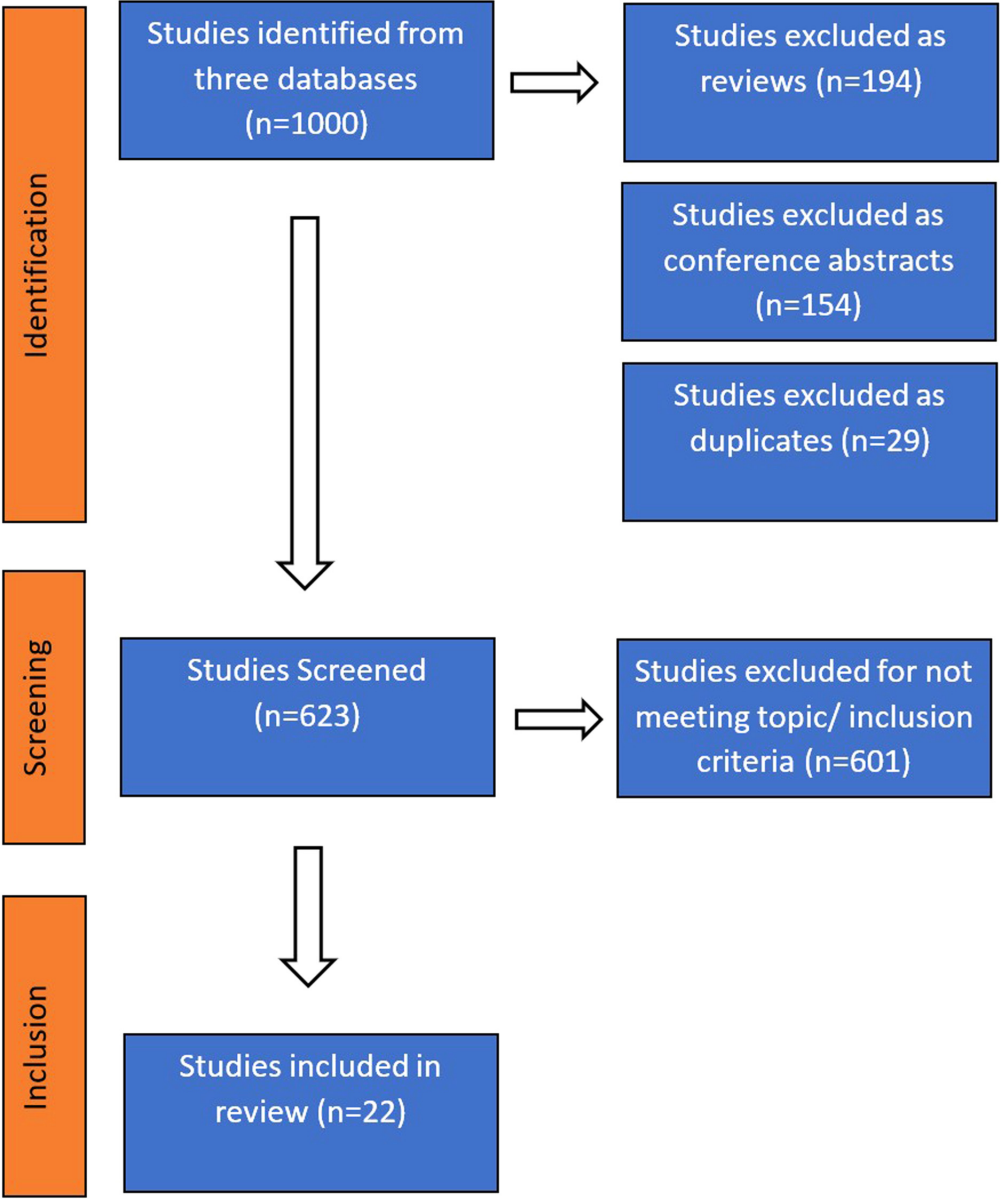
PRISMA flowchart.

The process of the literature search is summarized in Figure 1 as a PRISMA flowchart. From the 3 databases, n = 1000 articles were identified, of which 194 reviews, 154 conference proceedings/abstracts, and 29 duplicate articles were excluded, leaving 623 articles of the original 1000 to be screened further. A further 601 articles were excluded for not matching inclusion criteria, either as a consequence of not including immersive VR or AR technologies, or failing to address medical communication applications. Therefore, 22 relevant articles were ultimately reviewed. With PubMed database serving as the primary search, Web of Science and Embase only added one unique article each to the final inclusions. The 22 identified articles were published between 2012 and 2022, with 13 (59%) published most recently in 2021 to 2022; 14 employed cohort/cross-sectional design,^15-28^ 4 were case series/report,^29-32^ and 4 were randomized control trials (RCTs).^33-36^

## Results

All study characteristics are summarized in Table 1. It is worth noting that only 2 studies had a longitudinal design, measuring results at 2 or more different points in time.^15,36^ Sample size ranged from n = 2.^30,31^ to n = 165.^17^

**Table 1.** Summary of Study Characteristics.

Article	Year	Study design	Population and sample	Technology	Theme of results
Real et al^15^	2017	Cohort, Longitudinal	24 postgraduate pediatric residents	VR	Doctor–Patient Communication
Real et al^16^	2022	Cohort	22 clinicians deployed at 8 institutions	VR	Doctor–Patient Communication, Tolerability
Maloca et al^17^	2022	Cross-sectional	165 surveys from children aged 12-18	VR	Tolerability
Hara et al^18^	2021	Cohort	13 nursing educators and 30 nursing students	VR	Tolerability
Mill et al^19^	2021	Cohort	3 instructors, 53 medical students, 7 patient surveys	AR	Team Communication, Tolerability
Wright et al^20^	2020	Cross-sectional	50 surgical patients and 19 postgraduate neurosurgical residents in a single institution	VR	Doctor–Patient Communication
Yoon et al^21^	2021	Cohort	31 nurse trainees	AR	Doctor–Patient/Team Communication, Feasibility
Ma et al^22^	2021	Cohort	138 nursing students from 2 universities	VR	Empathy
Kim et al^23^	2021	Cross-sectional	21 nursing students	VR	Empathy
Diaka et al^24^	2021	Cross-sectional	Interviews 39 stakeholders—10 health center nurses, 5 hospital doctors, 11 patients, and 13 others	AR	Team Communication
McLaughlin et al^25^	2021	Cohort	10 medical students	VR	Empathy
Zahl et al^26^	2018	Cohort	23 volunteer medical students	AR	Team Communication, Feasibility
Kenngott et al^27^	2022	Cross-sectional	57 medical students, 48 surgeons, and 53 nurses	VR	Team Communication
Chang et al^28^	2020	Cohort	20 residents teaching 32 patients	VR	Doctor–Patient Communication
Phan et al^29^	2022	Case Series	46 SEEG patients	VR	Doctor–Patient Communication
Lin et al^30^	2013	Case Series	2 surgeons	VR	Doctor–Patient/Team Communication
Alexandrova et al^31^	2012	Case Series	2 scenarios	VR	Doctor–Patient Communication, Feasibility
Mamone et al^32^	2020	Case Report	Case report	AR	Doctor–Patient Communication, Feasibility
Real et al^33^	2017	RCT	24 postgraduate pediatric residents in intervention group, 21 in control group	VR	Doctor–Patient Communication
Perin et al^34^	2021	RCT	33 patients—11 per group	VR	Doctor–Patient Communication
Sapkaroski et al^35^	2022	RCT	70 trainee practitioners and 9 practitioners	VR	Doctor–Patient Communication
Liaw et al^36^	2020	RCT	120 undergraduate medical and nursing students	VR	Doctor–Patient/Team Communication

Abbreviations: VR, virtual reality; AR, augmented reality; RCT, randomized control trial.

### Themes

Articles on VR (n = 17) outnumbered articles on AR (n = 5). Similarly, more articles commented only on doctor–patient communication (11/22), compared to commenting only on intrateam communication (4/22), while 3 of 22 articles commented on both simultaneously. Six of the aforementioned articles additionally commented on tolerability or feasibility of their respective interventions,^16,19,21,26,31,32^ while a further 2 studies commented solely on tolerability.^17,18^ A final 4 of 22 articles focused on measuring empathy response after VR/AR intervention and were grouped in a separate category.^22,23,25,35^

### Interventions

VR-based studies contained a large variety of interventions—all with the aim of improving medical communication. Different intervention types included: 4 of 17 studies using VR to simulate a patient encounter,^15,31,33,36^ 4 of 17 implementing a VR teaching curriculum,^16,23,33,35^ 2 of 17 providing a VR video game experience,^18,22^ 4 of 17 using VR to educate patients,^20,29,30,34^ 2 of 17 using the headsets to view video recordings,^17,25^ and 3 of 17 using headsets to view 3D models. Interventions involving 3D model viewing were mostly associated with patient education,^28-30^ or facilitation of faster and easier preoperative planning.^27^ Other articles with interventions targeted at enhancing patient education achieved so by attempting to streamline informed consent.^20,34^ In some studies, the VR intervention was supplemented by other training methods like workshops, such as in those of Real et al.^16^ and Kim et al.^23^ Articles that assessed empathetic response mainly used simulation interventions, for example, a VR video game and simulation or recording of patient consultations, attempting to place the user in the patient's perspective.

There was more homogeneity with AR interventions: 4 of 5 articles used AR in live streaming, video recording, or remote communication purposes, and the remaining study described a wound projection intervention for enriched doctor–patient communication.^32^ Across both VR and AR, 10 study interventions were deployed in a live clinical environment, while the remaining 12 functioned in a training context. Moreover, only 4 studies compared VR/AR with one or more other interventions outside of VR/AR.^26,34-36^

**Figure 2.**
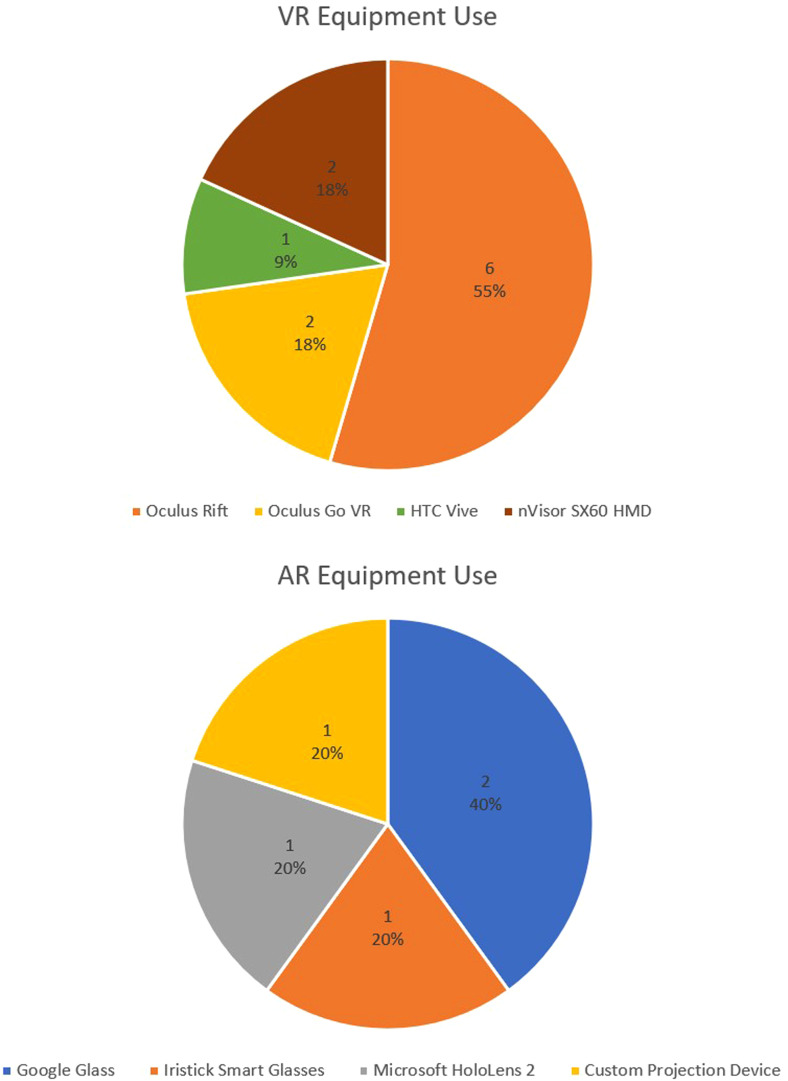
Distribution of equipment used in virtual reality/augmented reality (VR/AR) interventions.

Where specified, HMDs used in the VR-oriented studies include 6 uses of the Oculus Rift, 2 uses of the Oculus Go VR, one use of the HTC Vive, and 2 uses of the nVisor SX60 HMD. Meanwhile, AR-oriented studies listed a single Microsoft HoloLens 2 use, 2 Google Glass uses, a single Iristick Smart Glasses use, and 1 article which used a custom AR projection apparatus. The distribution of equipment used is displayed in Figure 2.

### Measures

Some studies employed different measures to gain multiple results, such as one quantitative measure to assess communication and another qualitative measure to assess participant opinions on the intervention.

There were 15 studies that used questionnaires across the cohort, the large majority of which were self-report. These questionnaires comprised a mix of quantitative and qualitative items, and 8 of them were standardized, validated questionnaires that had been previously used in other studies (eg, Spatial Presence Experience Scale^22^ and Video Review Assessment Effectiveness Scale).^26^ Further nonempirical measures included interviews, which were used in 3 studies,^23-25^ retrospective observation of VR use in clinical cases,^29^ and simple observations about the feasibility of a VR training curriculum in medical schools.^31^

Only 4 studies used empirical measures, including 2 RCTs. One RCT measured vaccine refusal rate as a measure of doctor–patient communication,^33^ while the other measured objective patient understanding of a treatment procedure.^34^ The other 2 studies recorded number and success of referrals stemming from the use of an AR intervention^24^ and gauged the feasibility of an AR projection device, determined through various empirical measures of accuracy.^32^ Of all studies, only 9 collected baseline or control measurements for comparison to assess the relative performance of interventions.

### Effect of Interventions

Results regarding intervention efficacy in enhancing communication were mostly positive across all types of communication. This applied to both VR and AR solutions.

In one RCT,^33^ a 9.3% reduction in vaccine refusal rate was measured, which aligns with the VR intervention's purpose. Another RCT found that objective comprehension of a treatment procedure by patients was higher in the VR intervention group, compared with non-immersive 3D display and 2D display groups.^34^ All 4 studies addressing empathy found that VR increased empathy response, including the third RCT in which a 5% increase in empathy response was seen.^35^ The only RCT in which the VR intervention did not improve on the control was Liaw et al,^36^ which found VR simulations of patient encounters to be of equal efficacy to live simulations when training clinician communication skills. However, this result was not framed to stigmatize VR but to propose it as a valid substitute for less efficient live simulations.

The only study in which the intervention was found to be ineffective in improving doctor–patient communication^21^ concluded that live streaming of clinical encounters using AR glasses only made it more difficult for students viewing the stream to identify communication skills and patient condition, also finding this system to be plagued with technical issues such as motion blur and vague audio transmission.

Other studies discussing feasibility and tolerability showed mixed results, with common difficulties being dizziness, sickness, and technical issues. For example, audio and visual quality criticisms were expressed by Mill et al^19^ Real and colleagues^16^ stated that 14% to 23% of clinicians experienced tolerability issues such as dizziness and eye strain. Similarly, Maloca et al^17^ found VR to be highly intolerable, with 54.89% of participants experiencing heavy symptoms and only 11.28% experiencing no symptoms at all. Conversely, Hara et al^18^ reported that participants had minimal tolerability problems with a VR game, and participants in the study of Zahl et al^26^ preferred the visual and audio quality of its Google Glass recording compared to a static camera recording. Furthermore, one study^32^ concluded that their custom AR projection device demonstrated enough accuracy to be classed as feasible for clinical use.

### Logic Model

**Figure 3.**
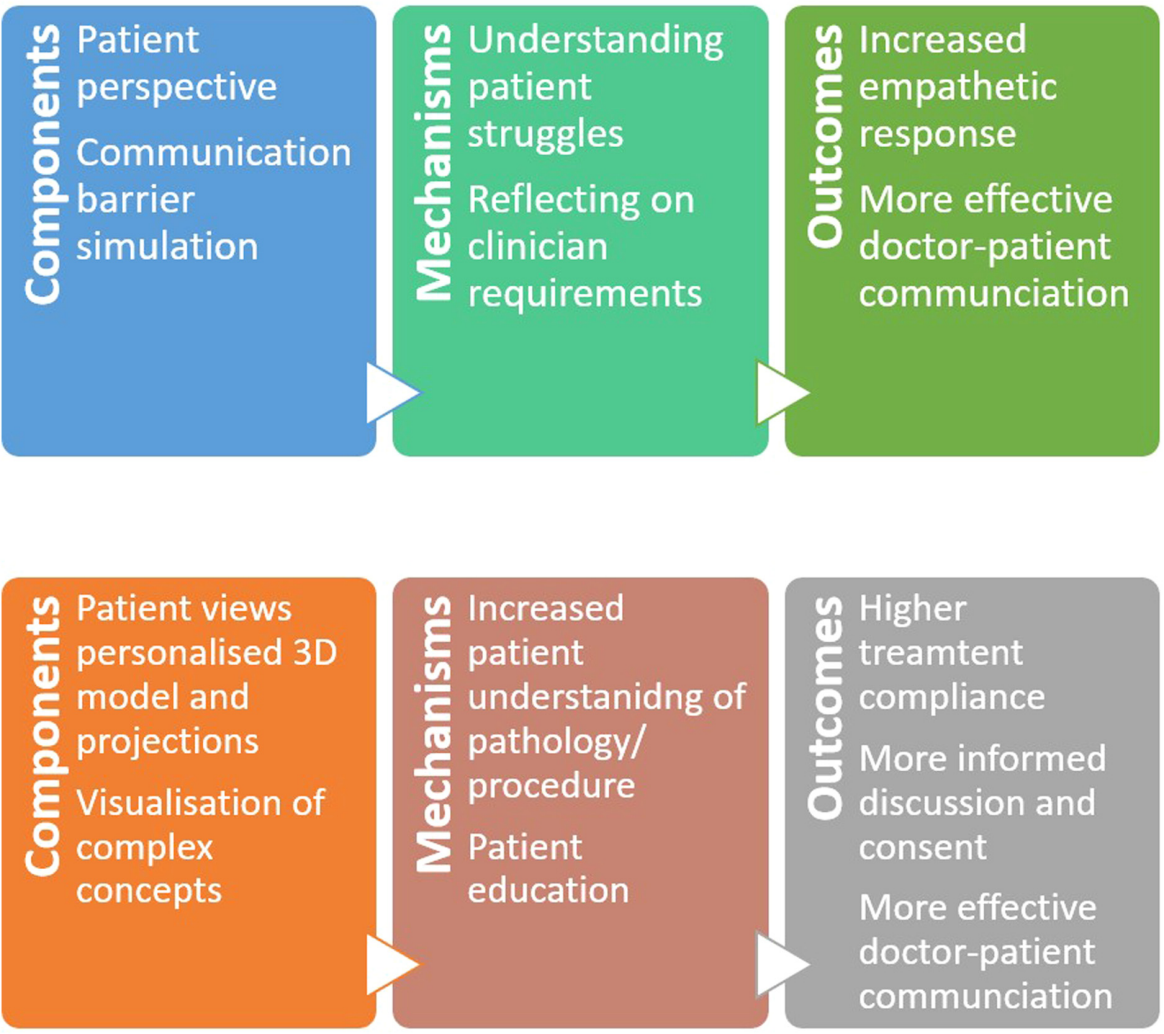
Logic model for virtual reality/augmented reality (VR/AR) (top), and logic model for 3D visualization technologies including VR, AR, and 3D printing (bottom).

The logic model shown in Figure 3 describes the mechanisms through which the interventions explored in this review achieved their effects. Studies focusing on empathy response placed the clinician in a consultation from the patient's perspective, while simulating any impairments (eg, deafness) or troubles they may experience through a 360° video or rendered video game. Subsequently, the clinician can more intimately understand the patient’s experience during an interaction, and what they could personally change about their behavior to show empathy. This can then result in more considerate and effective communication seen when clinicians show increased empathetic response. Some VR studies^20,29,30,34^ and an AR study^35^ were able to enhance patient education and the informed consent process by allowing patients to view relevant, individualized 3D models (VR) or wound projections (AR). This evidently increases patient understanding and provides a visual route for learning. Deductively, this can encourage the patient to remain adherent to treatment and engage in more informed dialogue about their condition with clinicians. The latter logic model is presented together with a previous one^14^ relating to 3D printing, merging considerations of both 3D visualization technologies, and showcasing similar functions between them. An external study^37^ reinforcing this resemblance between VR/AR and 3D printing showed that supplementing the VR experience with haptic feedback through peripherals (eg, Oculus Touch) can increase immersion and, therefore, amplify its effect, mirroring the haptic experience 3D-printed models provide.

## Discussion

In this review, the imbalance between the number of VR studies compared to AR studies is immediately clear. The overall limited quantity of evidence is amplified for AR, with only 5 studies identified. More research into the use of AR interventions specifically is required to form valid conclusions.

In general, communication as an abstract concept is difficult to quantify and operationalize. Quantitative measures of communication can have empirical merit, but they can be considered simplistic and reductionist when attempting to measure multifactorial human interactions. Hence, qualitative measures appear to be more suitable, in order to gain a more complete understanding of communicative processes. It should also be noted that communication style evolves over time. Contextual circumstances, such as during the Covid-19 pandemic when strict social distancing measures were in place, prompted innovative use of VR and AR in healthcare. Clinical staff and educators might not be adapting fast enough to convey lessons and ideas through this new medium. Furthermore, one key characteristic of patient–physician communication both in general practice and clinical care is empathy, which has been indicated as “the backbone of the patient–physician relationship”^38^ and defined as “the ability to understand the personal experience of the patient without bonding with them”.^39^

Most studies in this review gathered self-report data from questionnaires and interviews, which may introduce social bias into the results. An interview style also poses a host of researcher and confirmation biases, through potential leading questions and agenda promotion. Therefore, future research should focus on qualitative measures for a holistic evaluation of communication but take actions to minimize innate biases from interviews (such as using third party or multiple interviewers), or gather data from external observation performed by a designated panel of experts, as utilized in Sapkaroski et al^35^ and Liaw et al.^36^

Although most studies were published recently, the VR/AR space is so rapidly evolving that the 7 studies published prior to 2021 can be perceived to use outdated equipment and software, which weakens a substantial portion of the small body of evidence identified. Furthermore, the studies published recently are not guaranteed to be representing the current standard of technology to its full potential. More studies using modern VR/AR solutions are needed to expand the evidence presented here. There should also be an attempt to develop specialized, tailored software since many of the studies presented use general-purpose tools such as previously published video games, simulation software, and video streaming applications.

Only 4 RCT studies were identified in the search, and findings from the case series/report offer lower quality evidence. For example, Phan et al^29^ recorded data retrospectively from clinical records, which entails a lack of control over variables and dependence on the accuracy of the written record. Consequently, a deficiency in a high standard of evidence calls for more RCTs on this topic in the future.

Studies containing small samples produce results that can only be considered proof-of-concept and ungeneralizable to a wider population. Sample characteristics should also be considered critically, with most studies involving clinicians all of a single sex, or clinicians all in the same stage of training, again limiting generalizability. For example, Yoon et al^21^ recruited a sample of 74% female nurses, and Sapkaroski et al^35^ recruited solely first-year medical students. However, strength in some studies is represented by the attempt to gain a more extensive understanding of participants by surveying their level of past experience with VR/AR, allowing the sorting and weighing of results. Future studies should gather data from larger populations with matched baseline characteristics, to mitigate any extraneous variables.

Rigorous assessment of the technology is important to unravel its role in communicative processes, but in some instances, these observations may have been confounded or diluted. For example, Real et al^16^ included a supplementary communication workshop alongside the VR intervention, and the study of Wright et al^20^ is similar in that some participants took the intervention on a flat, non-immersive display, while others experienced immersive VR—both instances potentially confounding the results. A focused approach to interventions must be used in prospective studies, preferably with a control group and randomized design. Furthermore, with only 3 studies applying additional interventions outside of VR/AR, more multi-intervention research could be helpful in deducing how VR/AR interventions perform compared to other methods within the same framework.

Only 10 studies investigated their intervention's effect within a real clinical setting. Studies conducted in a controlled lab environment hold less external validity to reflect real-life results or mundane realism, especially in a situation as dynamic as the clinic. Generalizability to real-world applications can improve with more studies based in clinical settings.

Only 2 studies^15,36^ monitored the effect on communication over time (more than one point). This is extremely important as it auditions the intervention in a realistic scenario, where participants adjust and adapt appropriately. It gives more opportunity for more data to be gathered, and for temporal changes and patterns to be identified. Despite the study of Real et al^15^ being longitudinal in nature, it is still limited in that only 2 points in time were measured, over a relatively short one-month period. More longitudinal research, across a longer period of time, is a crucial component of gathering a valid wealth of evidence applicable to a realistic setting.

## Limitations

Despite having searched on 3 main databases, it is possible that some relevant articles exist on other databases. The focus of this scoping review is to map the landscape of current evidence on this topic, and while acknowledging possible biases (eg, confirmation bias in interviews) we did not measure the risk of bias systematically.

## Conclusion

This scoping review identified a gap in literature surrounding VR/AR usage and their effects on medical communication. The body of evidence is not only small in quantity but also superficial, particularly in the case of AR. Although there is unanimously positive sentiment toward the role of immersive VR and AR in improving medical communication, it is difficult to definitively conclude that their use is viable in current clinical applications, without more robust evidence. The apparent motion sickness and reliability limitations are also hurdles to overcome before widespread clinical adoption.

## References

[1] Dascal J Reid M IsHak WW , et al. Virtual reality and medical inpatients: a systematic review of randomized, controlled trials. Innov Clin Neurosci. 2017;14:14-21. Accessed August 20, 2022. https://www.ncbi.nlm.nih.gov/pmc/articles/PMC5373791/28386517 PMC5373791

[2] Ventura S Brivio E Riva G Baños RM . Immersive versus non-immersive experience: exploring the feasibility of memory assessment through 360° technology. Front Psychol. 2019;10. doi:10.3389/fpsyg.2019.02509 PMC6868024 31798492

[3] Sandrone S Carlson C . Future of neurology & technology: virtual and augmented reality in neurology and neuroscience education applications and curricular strategies. Neurology. 2021;97:740-4. doi:10.1212/WNL.0000000000012413 34187858

[4] Caria M Sara G Todde G Polese M Pazzona A . Exploring smart glasses for augmented reality: a valuable and integrative tool in precision livestock farming. Animals (Basel). 2019;9:903. doi:10.3390/ani9110903 31683920 PMC6912545

[5] Haskard Zolnierek KB DiMatteo MR . Physician communication and patient adherence to treatment. Med Care. 2009;47:826-34. doi:10.1097/mlr.0b013e31819a5acc 19584762 PMC2728700

[6] Wanzer MB Booth-Butterfield M Gruber K . Perceptions of health care providers’ communication: relationships between patient-centered communication and satisfaction. Health Commun. 2004;16:363-84. doi:10.1207/s15327027hc1603_6 15265756

[7] Doyle C Lennox L Bell D . A systematic review of evidence on the links between patient experience and clinical safety and effectiveness. BMJ Open. 2013;3:e001570. doi:10.1136/bmjopen-2012-001570 PMC3549241 23293244

[8] Thiedke CC . What do we really know about patient satisfaction? Fam Pract Manag. 2007;14:33-6. 17294978

[9] NHS. How good communication skills benefit patients, service users and people affected by cancer, and those important to them. Health Education England. March 22, 2022. Accessed January 27, 2023. https://www.hee.nhs.uk/our-work/cancer-diagnostics/cancer-communications-resource-hub/patient/how-good-communication-skills-benefit-patients-service-users-people-affected#:∼:text=Good%20communication%20skills%20have%20a

[10] Public Health England. *Public Health Outcomes Framework 2019-*2022. 2019. Accessed January 23, 2023. https://assets.publishing.service.gov.uk/government/uploads/system/uploads/attachment_data/file/862264/At_a_glance_document2.pdf

[11] Cieślik B Mazurek J Rutkowski S Kiper P Turolla A Szczepańska-Gieracha J . Virtual reality in psychiatric disorders: a systematic review of reviews. Complement Ther Med. 2020;52:102480. doi:10.1016/j.ctim.2020.102480 32951730

[12] Botella C Fernández-Álvarez J Guillén V García-Palacios A Baños R . Recent progress in virtual reality exposure therapy for phobias: a systematic review. Curr Psychiatry Rep. 2017;19:42. doi:10.1007/s11920-017-0788-4 28540594

[13] Dellazizzo L Potvin S Luigi M Dumais A . Evidence on virtual reality–based therapies for psychiatric disorders: meta-review of meta-analyses. J Med Internet Res. 2020;22:e20889. doi:10.2196/20889 32812889 PMC7468638

[14] Traynor G Shearn AI Milano EG , et al. The use of 3D-printed models in patient communication: a scoping review. J 3D Print Med. 2022;6:13-23. doi:10.2217/3dp-2021-0021 35211330 PMC8852361

[15] Real FJ DeBlasio D Ollberding N , et al. Resident perspectives on communication training that utilizes immersive virtual reality. Educ Health (Abingdon). 2017;30:228. doi:10.4103/efh.efh_9_17 29786025

[16] Real FJ Hood AM Davis D , et al. An immersive virtual reality curriculum for pediatric hematology clinicians on shared decision-making for hydroxyurea in sickle cell anemia. J Pediatr Hematol Oncol. 2022;44:e799-803. doi:10.1097/mph.0000000000002289 35319512 PMC8943226

[17] Maloca PM Williams EA Mushtaq F , et al. Feasibility and tolerability of ophthalmic virtual reality as a medical communication tool in children and young people. Acta Ophthalmol. 2022;100:e588-97. doi:10.1111/aos.14900 33988309 PMC9290670

[18] Hara CYN Goes FDSN Camargo RAA Fonseca LMM Aredes NDA . Design and evaluation of a 3D serious game for communication learning in nursing education. Nurse Educ Today. 2021;100:104846. doi:10.1016/j.nedt.2021.104846 33751998

[19] Mill T Parikh S Allen A , et al. Live streaming ward rounds using wearable technology to teach medical students: a pilot study. BMJ Simul Technol Enhanc Learn. 2021;7:494-500. doi:10.1136/bmjstel-2021-000864 PMC8154297 35520979

[20] Wright JM Raghavan A Wright CH , et al. Back to the future: surgical rehearsal platform technology as a means to improve surgeon-patient alliance, patient satisfaction, and resident experience. J Neurosurg. Published online October 2020:1-8. doi:10.3171/2020.6.jns201865 33096533

[21] Yoon H Kim SK Lee Y Choi J . Google glass-supported cooperative training for health professionals: a case study based on using remote desktop virtual support. J Multidiscip Healthc. 2021;14:1451-62. doi:10.2147/jmdh.s311766 34168458 PMC8216757

[22] Ma Z Huang KT Yao L . Feasibility of a computer role-playing game to promote empathy in nursing students: the role of immersiveness and perspective. Cyberpsychol Behav Soc Netw. 2021;24:750-5. doi:10.1089/cyber.2020.0371 33989057

[23] Kim HY Lee JH Lee EH . Virtual experience of perioperative patients: walking in the patients’ shoes using virtual reality and blended learning. Int J Environ Res Public Health. 2021;18:6457. doi:10.3390/ijerph18126457 34203661 PMC8296282

[24] Diaka J Van Damme W Sere F Benova L van de Put W Serneels S . Leveraging smart glasses for telemedicine to improve primary healthcare services and referrals in a remote rural district, Kingandu, DRC, 2019-2020. Glob Health Action. 2021;14. doi:10.1080/16549716.2021.2004729 PMC8667913 34889718

[25] McLaughlin N Rogers J D’Arcy J Gormley G . “Sorry doctor….I didn’t hear that….”: phenomenological analysis of medical students’ experiences of simulated hearing impairment through virtual reality. BMJ Simul Technol Enhanc Learn. 2021;7:207-15. 10.1136/bmjstel-2020-000683 PMC8936938 35516833

[26] Zahl DA Schrader SM Edwards PC . Student perspectives on using egocentric video recorded by smart glasses to assess communicative and clinical skills with standardised patients. Eur J Dent Educ. 2018;22:73-9. doi:10.1111/eje.12217 27380732

[27] Kenngott HG Pfeiffer M Preukschas AA , et al. IMHOTEP: cross-professional evaluation of a three-dimensional virtual reality system for interactive surgical operation planning, tumor board discussion and immersive training for complex liver surgery in a head-mounted display. Surg Endosc. 2022;36:126-34. doi:10.1007/s00464-020-08246-4 33475848 PMC8741674

[28] Chang SL Kuo MJ Lin YJ , et al. Virtual reality informative aids increase residents’ atrial fibrillation ablation procedures-related knowledge and patients’ satisfaction. J Chin Med Assoc. 2020;84:25-32. doi:10.1097/jcma.0000000000000464 PMC12966046 33230060

[29] Phan TN Prakash KJ Elliott RJS , et al. Virtual reality–based 3-dimensional localization of stereotactic EEG (SEEG) depth electrodes and related brain anatomy in pediatric epilepsy surgery. Childs Nerv Syst. 2022;38:537-46. doi:10.1007/s00381-021-05403-5 34718866

[30] Lin Q Xu Z Li B , et al. Immersive virtual reality for visualization of abdominal CT. Proc SPIE Int Soc Opt Eng. 2013;8673, doi:10.1117/12.2008050 PMC3877248 24386547

[31] Alexandrova IV Rall M Breidt M , et al. Enhancing medical communication training using motion capture, perspective taking and virtual reality. Stud Health Technol Inform. 2012;173:16-22. Accessed July 24, 2022. https://pubmed.ncbi.nlm.nih.gov/22356950/. 22356950

[32] Mamone V Fonzo MD Esposito N Ferrari M Ferrari V . Monitoring wound healing with contactless measurements and augmented reality. IEEE J Transl Eng Health Med. 2020;8:1-12. doi:10.1109/jtehm.2020.2983156 PMC7198047 32373400

[33] Real FJ DeBlasio D Beck AF , et al. A virtual reality curriculum for pediatric residents decreases rates of influenza vaccine refusal. Acad Pediatr. 2017;17:431-5. doi:10.1016/j.acap.2017.01.010 28126612

[34] Perin A Galbiati TF Ayadi R , et al. Informed consent through 3D virtual reality: a randomized clinical trial. Acta Neurochir (Wien). 2021;163:301-8. doi:10.1007/s00701-020-04303-y 32242272

[35] Sapkaroski D Mundy M Dimmock MR . Immersive virtual reality simulated learning environment versus role-play for empathic clinical communication training. J Med Radiat Sci. 2022;69:56-65. doi:10.1002/jmrs.555 34706398 PMC8892424

[36] Liaw SY Ooi SW Rusli KDB Lau TC Tam WWS Chua WL . Nurse-physician communication team training in virtual reality versus live simulations: randomized controlled trial on team communication and teamwork attitudes. J Med Internet Res. 2020;22:e17279. doi:10.2196/17279 32267235 PMC7177432

[37] Sapkaroski D Baird M McInerney J Dimmock MR . The implementation of a haptic feedback virtual reality simulation clinic with dynamic patient interaction and communication for medical imaging students. J Med Radiat Sci. 2018;65:218-25. doi:10.1002/jmrs.288 30006966 PMC6119726

[38] Derksen F Bensing J Lagro-Janssen A . Effectiveness of empathy in general practice: a systematic review. Br J Gen Pract. 2013;63:e76-84. doi:10.3399/bjgp13X660814 23336477 PMC3529296

[39] Moudatsou M Stavropoulou A Philalithis A Koukouli S . The role of empathy in health and social care professionals. Healthcare. 2020;8:26. doi:10.3390/healthcare8010026 32019104 PMC7151200

